# Effects of night-float shifts on cognitive function among radiology residents

**DOI:** 10.1007/s10140-024-02269-3

**Published:** 2024-07-24

**Authors:** Carl C. Flink, Robert E. Hobohm, Bin Zhang, Deborah L. Jacobson, Eric B. England

**Affiliations:** 1 Department of Radiology, University of Cincinnati Medical Center, CARE/Crawley Building, Suite E-870, 3230 Eden Avenue, Cincinnati, OH 45267 USA; 2 Division of Biostatistics and Epidemiology, Department of Pediatrics, Cincinnati Children’s Hospital Medical Center, Cincinnati, OH USA; 3 Division of Urology, Department of Surgery, University of Utah Medical Center, Salt Lake City, Utah USA

**Keywords:** Radiology, Shift work schedule, Internship and residency, Cognition, Actigraphy

## Abstract

**Background:**

Many radiology programs utilize a night-float system to mitigate the effects of fatigue, improve patient care, and provide faster report turnaround times. Prior studies have demonstrated an increase in discrepancy rates during night-float shifts.

**Objectives:**

This study was performed to examine the effects of night-float shift work on radiology resident cognition. We hypothesized that there would be diminished cognitive function on testing following night-float shifts when compared to testing following day shifts.

**Methods:**

Diagnostic radiology residents in their second to fifth years of residency at a single institution were recruited to participate in this pilot study. Cognitive function was evaluated using the Lumosity Neurocognitive Performance Tests (NCPT), standardized performance tests that provide real-time, objective measurements of cognitive function. Study participants completed the NCPT in 5 sessions following 5 consecutive day shifts to evaluate their baseline cognitive function. The tests were re-administered at the end of consecutive night-float shifts to assess for any changes. Sleep was objectively monitored using actigraphy devices worn around the wrist during all study weeks. Descriptive and summary statistics were performed.

**Results:**

23 prospectively recruited diagnostic radiology residents working night-float shifts took a mean 13.6 (± 5.1) neurocognitive performance tests during the study period. There was a statistically significant decline in 2 of the 6 cognitive tests administered, signifying a decrease in attention, speed, and complex reasoning ability. Night-float shifts were significantly longer than the day shifts and associated with a significantly higher study volume and cross-sectional study volume. Fitbit data demonstrated that there were no significant differences in level of activity while awake. However, participants slept significantly longer during day shifts.

**Conclusions:**

A sample of 23 radiology residents working night-float shifts demonstrated declines in attention, speed, and complex reasoning ability following sequential administration of standardized neurocognitive performance tests. While the sample size is small, these findings demonstrate the potential deleterious effects of night-float shift work and provide evidence to support further inquiry into this phenomenon.

**Supplementary Information:**

The online version contains supplementary material available at 10.1007/s10140-024-02269-3.

## Introduction

Between January 2005 and December 2021, delay in treatment was the third most common category of sentinel event reported by the Joint Commission, accounting for nearly 19% of all care management events over that timespan [1]. Within radiology, delays in diagnosis can arise from errors in perception and/or errors in interpretation. Analysis into sources of such errors suggests a number of contributing factors, one of which is physician fatigue. Multiple studies both inside and outside of the medical field demonstrate that fatigue leads to errors, with some suggesting fatigue can affect cognition more than even acute alcohol intoxication [2–4].

Traditional 24–36 h medical call shifts have been associated with alterations in mood, cognition, and psychomotor skills [5], which place both patients and physicians at risk for adverse events. While shifts of 24 h or more have become rare in radiology, there has been an increased emphasis on providing round-the-clock radiology coverage. Therefore, understanding and coping with physician fatigue is critical when optimizing overnight patient care.

Work hour limits, increasing clinical volumes and emphasis on turnaround times have resulted in the traditional 24–36-h call schedule giving way to night-float coverage and overnight shift work. However, recent studies have suggested that night-float shift work may have a deleterious effect on cognitive abilities [6–12]. Many of these studies have been limited by small sample sizes, variable controls, differences in overnight coverage, and conflicting results, making the cognitive impact of overnight shift work unclear. Greater evidence regarding the impact of night-float shift work on cognitive processes could ultimately reduce the propensity for diagnostic error, thereby improving patient care.

Recent studies involving overnight shift work among surgical residents demonstrate greater declines in cognitive ability than in psychomotor skills [8, 11, 13, 14]. Recent research has been performed exploring discrepancy rates of radiology residents [15] and attending radiologists [16] demonstrating increase in error rate during overnight shifts. Additional radiology specific research focused on eye tracking during overnight shifts has also been performed [17] and demonstrated an increase in false negatives and longer time to make the finding at the end of night-float shifts.

While many of these recent studies have had very practical endpoint measures (e.g., performance on laparoscopic simulators, report discrepancies) they are unable to evaluate decrement in individual components of cognition. For example, many of the studies within surgery were designed to assess deterioration of psychomotor skills [10, 11]. Using report discrepancies, while practical, fails to further differentiate errors related to perception, judgment, or lack of underlying knowledge. It also fails to account for compensatory measures used by residents (e.g., reviewing cases more slowly, greater reliance on outside resources, or consulting with other radiologists) that may mask cognitive decline. It would be naïve to postulate that attending physicians are not subject to a similar decline in cognitive performance, even if to a lesser degree, as has been previously suggested [13, 15].

Unfortunately, more rigorous evaluations of cognitive performance have their own set of drawbacks. Studies that have explored cognitive decline related to sleep deprivation and fatigue across numerous occupational fields have relied upon thorough assessments of cognition that are often costly [18], time consuming [3], and/or prone to the effects of learning from repeated examinations, thus limiting their effectiveness in repeated test measures.

It is for these reasons that we chose to utilize Lumosity’s NeuroCognitive Performance Test (NCPT), a web-based testing platform with computerized assessments, to examine the effects of night-float shift work on radiology resident cognition. Lumosity’s NCPT is a validated, normalized platform that provides an objective, practical means of assessing cognitive performance [19]. The web-based platform allows for convenient administration and addition of new testing sites. Our pilot study was performed to examine the effects of night-float shift work on radiology resident cognition. We hypothesized that there would be diminished cognitive function on testing following night-float shifts when compared to testing following day shifts.

## Methods

Institutional Review Board approval for this study was obtained and was compliant with the Health Insurance Portability and Accountability Act. Each subject provided written informed consent prior to the first NCPT. The resident radiologists were compensated $100 for participation in the study. A Fitbit® Charge 3 device was provided to each participant to track actigraphy data. Participants retained their Fitbit devices after completion of the study.

Volunteers for the study included third, fourth-, and fifth-year diagnostic radiology residents who perform night-float shift coverage as a part of their training at the University of Cincinnati. Prior to data collection participants were prompted to create an account with Lumosity for purposes of tracking and recording data. Personal data including age, sex, and post-graduate year was collected for purposes of scaling performance on the various assessments. All data was de-identified and analyzed in aggregate in accordance with Lumos Labs’ Privacy Policy.

For purposes of this study, we included residents who had a single week of night-float shift work or were on the first week of a two-week rotation of night-float shift work. Weekend night-float shifts had a duration of 15 h, from Sunday night to Monday morning (17:00 – 08:00) and were single events. Weekday night-float shifts had a duration of 11 h (21:00 – 08:00) and were consecutive events occurring nightly from Monday-Saturday nights. Residents were asked to complete the NCPT assessments within three hours of the conclusion of their night-float shift.

Of the 16 subtests available via the Lumos Lab’s NCPT, we focused on 6 tests that emphasized important subdomains in radiology resident competencies: Dual Search, Go/NoGo, Grammatical Reasoning, Memory Span, Object Recognition, and Trail Making. The Dual Search test asks subjects to identify a peripheral target letter among a cluster of letters and evaluates attention. The Go/NoGo test asks subjects to immediately react to a target stimulus while avoiding distractors, evaluating attention, speed, and impulsivity. The Grammatical Reasoning test asks subjects to evaluate the truth of statements regarding the position of shapes, evaluating logical reasoning and mental flexibility. The Memory Span test asks subjects to recall a randomized sequence of lighted circles, evaluating short term memory. The Object Recognition test asks subjects to recall whether an image was previously presented, evaluating memory. Finally, the Trail Making test asks subjects to connect numbers and letters in ascending order, evaluating mental flexibility and processing speed (Supplemental Table 1).

After creating an account, participants were prompted to complete the NCPT a minimum of two times in a one-month period in order to familiarize themselves with the software platform and assessment format. Our study control was completion of the assessment at the conclusion of 5 routine day shifts (08:00 – 17:00) over the course of a typical 5-day workweek (Monday-Friday). To mitigate confounding effects of circadian rhythm dysfunction and altered sleep cycles, participants were instructed that they were ineligible to take the baseline survey within five days of working a night-float shift. Participants then completed assessments. Additionally, to control for seasonal variations, we encouraged participants to complete the assessment within 30 days preceding or following their night-float shift as scheduling permitted. Participants then completed assessments following each consecutive night-float shift worked. Participants were asked to complete the NCPT assessments within three hours of the completion of each of their shifts.

After each assessment participants completed the Stanford Sleepiness Scale (to gauge subjective levels of fatigue) and answered questions regarding shift length, number of consecutive nights worked, hours of sleep in the last 24 h, hours of sleep during their shift, stimulant use (reference dose/serving provided), use of sleep aids or chronobiotics, volume of total studies read, and volume of cross-sectional studies read (e.g., CT, MRI, or ultrasound). The final component of the survey was the Patient Health Questionnaire-9, a validated assessment of changes in mood that might not meet criteria of depression.

Following completion of the assessment, a participant’s raw scores for the NCPT were tabulated for each of the six individual subtests under both the control and experimental conditions. The raw data for each assessment was scaled based upon normalized data sets to arrive at the performance for each subtest. These scores were then summed, and the same rank-based inverse normal transformation was applied to the subtest scores to compute a grand index score for the entire assessment. Average performance and standard deviations were calculated for both conditions, and t-tests were utilized to assess for significance. Random effects models were run to consider the correlation of scores for the same participant. Similar statistical analysis was performed on the individual subtests designed to elicit which aspects of cognition were affected more acutely and which were more resilient. All statistical analysis was performed with SAS Version 9.4 (SAS Institute Inc).

## Results

A total of 23 diagnostic radiology residents working at least 6 consecutive night-float shifts completed a mean 13.6 (± 5.1) neurocognitive performance tests while enrolled in the study. The mean Go/NoGo score, a measure of response time, was 20.3 points (± 26.9) higher after night-float shifts than day shifts (p = 0.02); in this test, the higher score indicates a longer (worse) response time after night-float shifts. The mean Grammatical Reasoning score was 1.8 points (± 2.3) lower after night-float shifts than day shifts (p = 0.02); in this test, a lower score indicates fewer correct answers (worse) after night-float shifts. Mean scores in Dual Search, Memory Span, and Trail Making were not significantly different between groups, while mean scores in Object Recognition, a test of memory, trended towards higher (better) after night-float shifts (p = 0.08; Table 1).

**Table 1:** Summary of test outcomes

Test Name	Mean Score*	SD	*p*-value*
Dual Search	0.50	1.9	0.39
Go/NoGo	-20.3	26.9	0.02
Grammatical Reasoning	1.8	2.4	0.02
Memory Span	0.32	1.1	0.35
Object Recognition	-0.51	0.92	0.08
Trail Making	-1344.2	4574.6	0.33

*Mean difference in daytime minus nighttime parameters on paired t-test

**Paired t-test

The results from the paired t-test were confirmed by running two random effects models that considered the correlation of scores from the same participant. The “Average” model, using the average score for one shift as the outcome, demonstrated significant differences in the Grammatical Reasoning score (p = 0.01) but not in other domains. The “Individual” model, using the daily score as the outcome, demonstrated significant differences in the Grammatical Reasoning (worse after night-float shifts, p = 0.002), Object Recognition (better after night-float shifts, p = 0.02), and Go/NoGo (worse after night-float shifts, p = 0.03) scores, but not in other testing domains (Supplemental Table 2).

Day shifts lasted a mean 9.5 h (± 1.4) during which residents read a mean 29.4 studies per shift (± 19.8), an average of 3.1 studies per hour. On average, 46.2% (13.6 studies ± 14.1) of the day shift studies were cross sectional. Night-float shifts lasted a mean 11.9 h (± 1.9) during which the residents read a mean 78.1 studies per shift (± 21.7), an average of 6.6 studies per hour. On average, 42.2% (33.0 studies ± 9.5) of the night-float shift studies were cross sectional.

Paired t-tests comparing shift parameters between day and night-float shifts demonstrated that the night-float shift was significantly longer than the day shift (p < 0.001) and was associated with a significantly higher study volume (p < 0.001). Residents slept a mean 6.4 h (± 1.0) at night during the day shifts versus 6.0 h (± 1.6) during the day on night-float shifts, a statistically significant reduction in hours of sleep (p = 0.002; Table 2). Results from the paired t-test were confirmed by random effects models (“Average” and “Individual”), with significant differences noted in shift length, study volume, cross sectional study volume, and hours slept in both models (Supplemental Table 3).

**Table 2:** Comparison of shift parameters between day and night shifts

Variable	Day		Night		Paired T-test		
	Mean	SD	Mean	SD	Mean Difference*	SD	*p*-value
Shift length (hours)	9.5	1.4	11.9	1.9	-2.5	1.1	<0.001
Consecutive nights worked	NA	NA	3.7	1.9	NA	NA	NA
Total study volume	29.4	19.8	78.1	21.7	-61.0	21.2	<0.001
Cross sectional study volume (CT, US, and MR)	13.6	14.1	33.0	9.5	-24.3	7.9	<0.001
Hours slept in 24-hour period	6.4	1.0	6.0	1.6	0.80	0.66	0.002

*Mean difference in daytime minus nighttime parameters on paired t-test

Multivariable regression models run for each subtest, adjusting for all shift parameters, demonstrated significantly higher Object Recognition scores (p < 0.001) and significantly lower Grammatical Reasoning scores (p = 0.02) after night-float shifts. There were no differences found in Dual Search, Go/NoGo, Memory Span, and Trail Making scores on multivariable regression analysis.

Fitbit data demonstrated that there were no significant differences in level of activity while awake between day and night-float shifts. However, participants slept significantly longer during day shifts (p = 0.02) and spent more time in bed (p = 0.02; Table 3). A participant survey demonstrated that the degree of sleepiness was higher during day shifts than night-float shifts (p < 0.0001) but there were no differences in mood or other qualitative parameters during the study period (Table 4).

**Table 3:** Comparison of activity and sleep data between day and night-float shifts

Activity	Mean Difference*	*p*-value
Calories burned	-154	0.21
Steps	-671	0.43
Distance	-0.32	0.45
Floors	2.2	0.34
Minutes sedentary	52	0.46
Minutes lightly active	-27	0.20
Minutes fairly active	-6	0.10
Minutes very active	1	0.85
Activity calories	-148	0.28
Sleep		
Minutes asleep	51	0.02
Minutes awake	8	0.06
Number of awakenings	6	0.02
Time in bed	58	0.02
Minutes REM sleep	11	0.08
Minutes light sleep	32	0.008
Minutes deep sleep	5	0.26

*Mean difference in daytime minus nighttime parameters on paired t-test

**Table 4:** Paired responses among residents surveyed after day and night-float shifts

Variable	*N*	Mean**	SD	*p*-value	
				Paired T-test	Signed Rank
Little interest or pleasure in doing things	15	8.0	29.9	0.32	0.56
Feeling down, depressed, or hopeless	15	7.2	22.6	0.24	0.56
Trouble falling or staying asleep, or sleeping too much	15	20.4	42.6	0.08	0.13
Feeling tired or having little energy	14	11.9	47.9	0.37	0.31
Poor appetite or overeating	15	7.4	34.0	0.41	0.74
Feeling bad about yourself or that you are a failure or have let yourself or your family down	15	0.15	22.0	0.98	0.5
Trouble concentrating on things, such as reading the newspaper or watching television	15	10.0	31.9	0.24	0.22
Moving or speaking so slowly that other people could have noticed. Or the opposite, being so fidgety or restless that you have been moving around a lot more than usual	15	-0.60	4.9	0.64	0.63
Thoughts that you would be better off dead or of hurting yourself in some way	15	-0.51	5.0	0.70	0.75
How difficult have these problems made it for you to do your work, take care of things at home, or get along with other people	15	6.5	15.7	0.13	0.15
Please indicate your degree of sleepiness*	15	2.0	0.98	< .0001	< .0001

* “Please indicate your degree of sleepiness” was treated and compared as continuous variable

**Mean of difference between day and night-float shifts (e.g. mean 8.0 signals that % of “yes” during day shifts was 8.0% higher than during night-float shifts)

## Discussion

Numerous previous studies have examined the effects of overnight shift work on cognition both within medicine [8–10, 13, 20–22] and outside medicine [3, 23–26]. The vast majority demonstrated decline in cognition that is greatest when testing higher levels of cognitive function. However, to our knowledge, no study to date has evaluated cognitive function in radiology residents. Instead, discrepancy rates have been used as a surrogate for cognition with a demonstrable increase in discrepancies during overnight shifts [16]. Additionally, eye tracking data has shown significant decrease in performance after overnight shifts [17]. While these studies are important and their results are a valuable addition to our knowledge of the challenges faced by radiologists working overnight shifts, we want to evaluate for decay in specific components of cognitive function that may help better understand the reason for mistakes in judgement seen above. Conversely, we also hoped to establish components of cognition that demonstrate resilience in the face of fatigue and shift work.

Our study was designed to evaluate individual components of cognitive performance in radiology residents when working daytime shifts compared to overnight shifts. Many residency programs have transitioned from the traditional 24 h call structure to a night-float system. Additionally, many departments have implemented overnight shift work for attending physicians. As such, we felt it important to better understand the impact this type of shift work has on the individual components of cognition. We hypothesized that in our resident cohort there would be a decline in cognitive performance as compared to daytime shifts, and we selected cognitive subtests specifically for their applicability to radiology. Our hypothesis proved at least partially correct as scores on the Go/NoGo test demonstrated a significantly longer response time and scores on the Grammatical Reasoning test demonstrated a significantly lower number of correct responses among residents performing night-float shift work.

Our study results demonstrate that radiology residents experience decreased cognition, as demonstrated by worsened scores on the Go/NoGo and Grammatical Reasoning tests, following night-float shiftwork. The Go/NoGo test is a measure of attention, speed, and impulse, all of which are crucial to the basic function of a radiologists. Diminished attentiveness may predispose residents to erroneous study interpretations, as reported in the literature, while increasing view time per case and gaze fixation. The Grammatical Reasoning test evaluates the highest levels of cognitive function including the ability to reason, form concepts, and solve problems using unfamiliar or novel information. Worsened performance in this area of cognition may predispose radiologists to errors when evaluating complex patient imaging. Performance on testing aimed at memory remained intact, however, which agrees with data from a large prior study [25].

An additional challenge of night-float shift work is disruption of the normal circadian rhythm, which can result in worsened quality and quantity of sleep [27]. Multiple prior studies have associated both lack of sleep [13, 21, 22, 28] and subjective fatigue [13] with worsened cognitive function. Our study provided objective (actigraphy) and subjective (survey) sleep data, demonstrating statistically significant increases in feelings of sleepiness and decreases in the ability to fall asleep during night-float shift work.

Given that fatigue has been show to drastically impact mood [29], we also hypothesized that we would see a measurable worsening of resident’s mood during night-float shift weeks. Somewhat surprisingly, no notable decrease in mood was noted in our study. The reason for this is unclear and may reflect culture of the residency program, work environment, or resident resilience in a rather small cohort.

There are several limitations to our study findings. First, we present findings from a pilot study limited to the residents in our department who worked night-float shift rotations. We also had suboptimal study response rate, including a small number of residents who performed their cognitive tests outside the prescribed study time windows. Therefore, the study was likely underpowered, increasing the chance of a type II error. Additionally, all participants were from a single institution with a specific work culture and studied at a particular timepoint. Therefore, findings may not be generalizable to the larger resident workforce. Finally, the NCPT was administered at a limited number of time points during the study period, and therefore does not reflect intra-shift fluctuations in performance. While previous data has shown a nadir in performance in the early morning hours [15, 24], it was not feasible to administer the tests at multiple timepoints given the night-float shift rotation workload.

In conclusion, sequential neurocognitive tests administered to 23 residents following day and night-float shift work demonstrated statistically significant declines in attention, speed, and complex reasoning ability following night-float shifts. There was a concomitant objective decline in sleep quantity and subjective increase in resident sleepiness during night-float shifts. While the sample size is small, these findings demonstrate the potential deleterious effects of night-float shift work and provide evidence to support further inquiry into this phenomenon. We hope to use the data from our pilot study to power a future multi-institutional study with much larger cohort to validate our findings and to evaluate the impact of long-term night-float shift on attending physician cognition [23]. Future studies would benefit from an increase in sample size and diversification of resident specialty. Other potential study directions include evaluating strategies to mitigate the effects of night shift work such as careful use of stimulants such as caffeine, planned use of chronobiotics such as melatonin, and circadian phase shifting, among others [27, 30]. In the interim, residency programs should outline strategies to compensate for those deficits to optimize patient care, minimize physician error, emphasize resident education, and facilitate staff scheduling practices.

## Supplementary Information

Below is the link to the electronic supplementary material.

Supplementary file1 (DOCX 26 KB)

## Data Availability

The authors confirm that the data supporting the findings of this study are available within the article.

## Abbreviation

NCPT: Neurocognitive Performance Test
