# Supplementary material for: Effects of night-float shifts on cognitive function among radiology residents
Source: Emerg Radiol. 2024 Jul 24;31(5):725–31. doi: 10.1007/s10140-024-02269-3 (PMC11436431; doi:10.1007/s10140-024-02269-3)
Supplement: Supplementary file 1 — Supplementary file1 (DOCX 26 KB) [file 10140_2024_2269_MOESM1_ESM.docx]

Supplemental Table 1. Subtest descriptions

| **Test Name** | **Test Domain(s)** | **Test Description** | **Test Measure** |
| --- | --- | --- | --- |
| **Dual Search** | Attention | A cluster of letters and a peripheral target letter are shown.  *  Subjects are asked to identify the peripheral letter or whether the cluster of letters were all the same. | Number of correct responses |
| **Go/NoGo** | Attention  Speed  Impulse | A target stimulus appears.  *  Subjects respond as quickly as possible when a target stimulus appears while avoiding response to distractors. | Mean response time |
| **Grammatical Reasoning** | Logical Reasoning Mental Flexibility | A square and a triangle are shown side by side.  *  Subjects are asked whether a statement about the shapes is true or false. | Number of correct responses minus number of incorrect responses |
| **Memory Span** | Memory | Circles in randomized spatial locations are lighted in a sequence.  *  Subjects recall the correct sequence | Number of correct trials before two consecutive errors during one sequence |
| **Object Recognition** | Memory | Images are presented.  *  Subjects make a forced choice response regarding whether an image was previously presented. | Number of correct responses |
| **Trail Making** | Mental Flexibility  Processing Speed | Numbers and letters are presented.  *  Subjects connect the numbers from smallest to largest, alternating between numbers and letters. | Completion time |

Supplemental Table 2. Paired t-test random effects models for test outcomes

| **Score** | **Average** | | | **Individual** | | |
| --- | --- | --- | --- | --- | --- | --- |
|  | **Estimate** | | **p-value** | **Estimate** | | **p-value** |
|  | **Day** | **Night** |  | **Day** | **Night** |  |
| Dual search | 42.8 | 41.9 | 0.41 | 42.7 | 42.3 | 0.56 |
| Go/NoGo | 417.2 | 435.4 | 0.16 | 415.6 | 433.2 | 0.03 |
| Grammatical Reasoning | 15.9 | 14.0 | 0.01 | 15.9 | 14.0 | 0.002 |
| Memory Span | 11.5 | 11.1 | 0.31 | 11.4 | 11.2 | 0.37 |
| Object Recognition | 18.0 | 18.4 | 0.10 | 17.9 | 18.5 | 0.02 |
| Trail Making | 26715 | 28144 | 0.35 | 26836 | 27875 | 0.46 |

Supplemental Table 3. Paired t-test random effect models for shift parameters

| **Variable** | **Average** | | | **Individual** | | |
| --- | --- | --- | --- | --- | --- | --- |
|  | **Estimate** | | **p-value** | **Estimate** | | **p-value** |
|  | **Day** | **Night** |  | **Day** | **Night** |  |
| Shift length (hours) | 9.5 | 12.0 | <0.001 | 9.4 | 12.0 | <0.001 |
| Total study volume | 25.5 | 82.2 | <0.001 | 28.0 | 81.7 | <0.001 |
| Cross sectional study volume (CT, US, and MR) | 11.4 | 34.6 | <0.001 | 12.4 | 34.7 | <0.001 |
| Hours slept in 24-hour period | 6.6 | 6.0 | 0.01 | 6.7 | 6.0 | 0.002 |
